# Deactivation of implantable cardioverter defibrillator in Japanese patients with end‐stage heart failure

**DOI:** 10.1002/joa3.12465

**Published:** 2020-11-24

**Authors:** Mayui Nakazawa, Tsuyoshi Suzuki, Tsuyoshi Shiga, Atsushi Suzuki, Nobuhisa Hagiwara

**Affiliations:** ^1^ Department of Cardiology Tokyo Women's Medical University Tokyo Japan; ^2^ Department of Clinical Pharmacology and Therapeutics The Jikei University School of Medicine Tokyo Japan

**Keywords:** deactivation, do not attempt resuscitation, end of life, heart failure, implantable cardioverter defibrillator

## Abstract

**Background:**

Despite the effectiveness of implantable cardioverter defibrillators (ICDs) in the prevention of sudden cardiac death, shock therapy causes patients to experience pain and psychological distress, which contradicts the purpose of palliative care. It is difficult to predict the time course for heart failure (HF) patients, unlike that for cancer patients. The aim of this study was to evaluate the deactivation status of ICD therapy in Japanese patients with end‐stage HF.

**Methods:**

We retrospectively studied 51 ICD patients who died due to worsening HF at Tokyo Women's Medical University Hospital from 2010 to 2019. The frequency of ICD therapy delivered before death and information about the discussion of deactivation and do not attempt resuscitation (DNAR) decisions were reviewed using medical charts.

**Results:**

Of 51 patients, 12 (24%) patients deactivated ICD therapy and seven patients underwent deactivation within 24 hours of a DNAR order. The median time from deactivation to death was 3 days (range, 0‐56). Of 39 patients with DNAR orders, 27 (69%) did not undergo deactivation. A relatively high proportion of patients (n = 14, 27%) experienced ICD shocks within 1 month of death. The frequency of electrical storms within 1 month of death was also high (n = 12, 24%).

**Conclusions:**

Our study showed that only one‐fourth of Japanese patients with end‐stage HF underwent deactivation of ICD therapy. A relatively high frequency of shock therapy was observed in the last month before death.

## Graphical abstract

Only one fourth of Japanese patients with end‐stage heart failure underwent deactivation of implantable cardioverter defibrillator (ICD) therapy. The frequency of ICD therapy within one month of death was higher than that prior to one month before death in patients with end‐stage heart failure, and some patients received shocks within 24 hours of death.

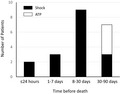

## 1 INTRODUCTION

An implantable cardioverter defibrillator (ICD) improves survival in patients at high risk for sudden cardiac death (SCD).^1^ Despite the effectiveness of ICDs in the prevention of SCD, shock therapy causes pain in patients and psychological distress for patients and family members who provide their care.^2^ This issue contradicts the purpose of palliative care in terminally ill patients.

For end‐stage patients with cardiovascular disease, especially heart failure (HF), deciding whether to deactivate ICD treatment, including shock, is an important medical and ethical problem. A recent pilot study of quality indicators for palliative care in Japanese patients with chronic HF reported that interdisciplinary discussion about ICD deactivation at the end of life was inadequate.^3^ In patients with end‐stage HF, not all treatments are discontinued, because the HF treatments themselves can improve symptoms. Furthermore, if the symptoms of HF patients worsen, patients improve promptly with acute treatment over the short term. This may cause a tendency to overestimate life expectancy rather than identify the patient's actual status.^4^ In practice, it is often difficult to predict the time course in advanced HF patients. For these reasons, physicians cannot discuss ICD deactivation with a patient and family members in advance or determine the appropriate timing for turning it off in the clinical setting.

Recently, there have been some reports regarding deactivation of ICD therapy in patients who were judged to have end‐stage diseases, including malignancy, infections, and chronic obstructive pulmonary disease as well as HF.^5^, ^6^, ^7^, ^8^, ^9^ However, there are few reports on the therapy status of ICD patients with end‐stage HF in Japan. The aim of this study was to evaluate the deactivation status of ICD therapy in ICD patients with end‐stage HF.

## 2 METHODS

### 2.1 Patients

**FIGURE 1:**
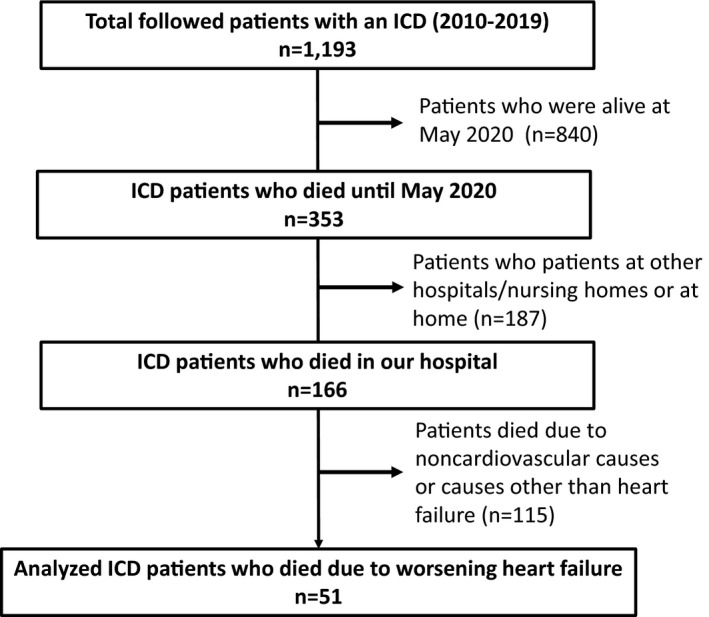
Flow diagram of the study patients

We conducted a retrospective observational study with 1193 consecutive Japanese patients who underwent ICD and were followed at Tokyo Women's Medical University Hospital from August 2010 to May 2019. We first searched the ICD implant patient databases. Then, we confirmed each patient's survival/death and cause of death by checking their medical records. We excluded ICD patients who were alive as of May 1, 2020 and patients who died at other hospitals/nursing homes or at home due to any cause of death because we could not obtain detailed information about the patients’ treatment and care processes before death. Among patients who died at our hospital, we excluded patients who died due to noncardiovascular causes or causes other than HF. Ultimately, we included 51 ICD patients who died due to worsening HF at our hospital in this analysis (Figure 1). End‐stage HF was defined as the presence of progressive or persistent severe signs and symptoms of HF despite the use of optimal medical or nonpharmacologic treatments.^10^, ^11^ The protocol was approved by the Institutional Review Board of Tokyo Women's Medical University (approval number 5442).

### 2.2 Data collection

Data on patient age, gender, underlying heart disease, comorbidities, laboratory results, concomitant medications, indication for ICD implantation, type of device, date of device implantation, and date of device deactivation were obtained from medical records and laboratory data. We also collected the following patient clinical information: reason and diagnosis leading to device deactivation, discussion about palliative care with an interdisciplinary team, discussion about deactivation, do not attempt resuscitation (DNAR) decisions, date of a DNAR order, and presence or absence of opioid therapy.

Data on ventricular tachyarrhythmia occurrences that required ICD interventions, including both shock and antitachycardia pacing (ATP), were retrieved from ICD interrogation reports stored on the disks in each device. Relevant event details and electrocardiograms were reviewed by two independent investigators (AS, TS). An electrical storm was defined as the occurrence of three or more separate episodes of VT or VF within 24 hours, regardless of the mode of termination.^12^

### 2.3 Statistical analysis

Summary data are presented as the number of patients and the means and standard deviations. Continuous variables were compared between groups with and without ICD deactivation using Student's *t* test and the Mann‐Whitney *U* test. Categorical variables were subjected to chi‐square analysis. Data analyses were performed using the JMP statistical software program (version 13, SAS Institute Inc).

## 3 RESULTS

### 3.1 Patients and DNAR order

**FIGURE 2:**
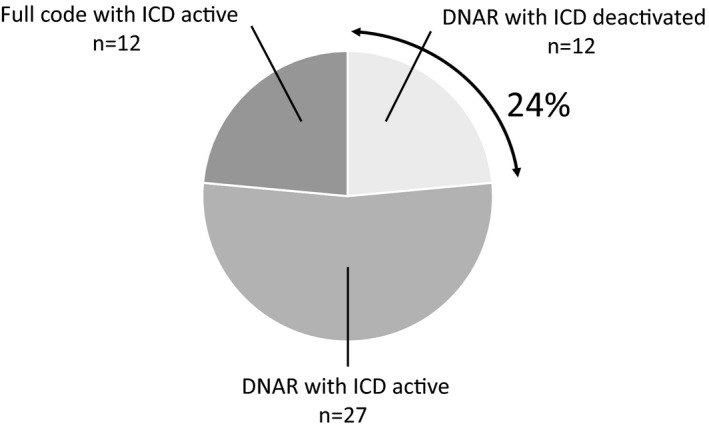
Patient and device status at the time of death. DNAR, do not attempt resuscitation; ICD, implantable cardioverter defibrillator

Among 51 ICD patients who died due to worsening HF in our hospital, 31 (61%) patients had implanted ICDs for the primary prevention of SCD. The median duration from device implantation to death was 69 months (range, 8‐228). In this study, 39 (76%) patients had a DNAR order (Figure 2). The time from the DNAR order to patient death had a median of 5 days (range, 0‐77).

### 3.2 Deactivation of ICD therapy

**FIGURE 3:**
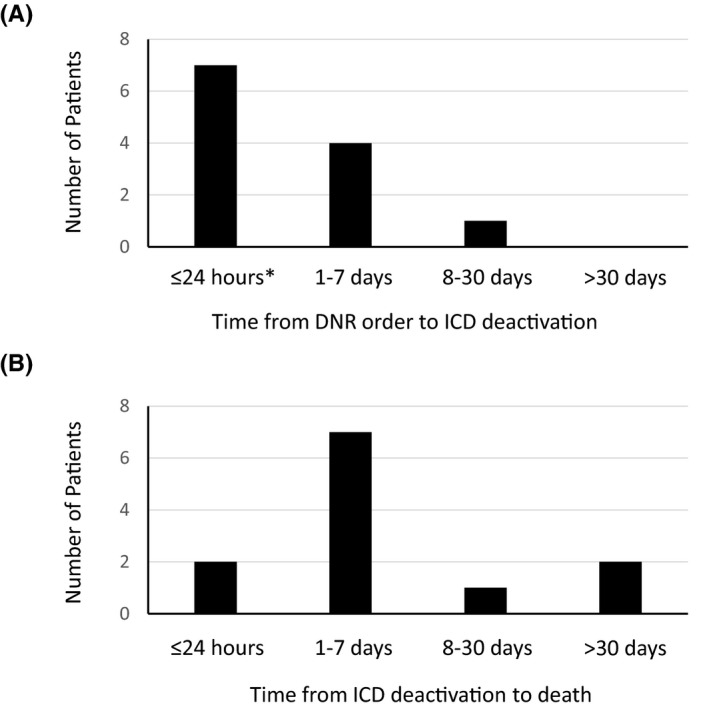
A, Time from do not attempt resuscitation (DNAR) order to implantable cardioverter defibrillator (ICD) deactivation and (B) time from ICD deactivation to death in patients with end‐stage heart failure. *Two patients who deactivated ICD therapy prior to DNAR decision

Deactivation of ICD therapy was performed in 12 (24%) patients (Figure 2). Among them, six patients received intravenous inotropes, one patient received an intra‐aortic balloon pump, and one patient received percutaneous cardiopulmonary support at the time of ICD deactivation. Seven (58%) patients underwent deactivation within 24 hours of the DNAR order, but some had more than 2 days pass between the DNAR order and deactivation (Figure 3). On the other hand, 27 patients did not undergo deactivation of the ICD despite the DNAR order, and the remaining 12 patients did not give a DNAR order. The characteristics and comparisons of patients with and without ICD deactivation are shown in Table 1. There were no significant differences in the baseline clinical and demographic characteristics, between the groups, except that the left ventricular ejection fraction was higher in patients with deactivation than in those without deactivation. Regarding treatments during the last hospitalization, the frequency of angiotensin‐converting enzyme inhibitor/angiotensin II receptor blocker use and beta blocker use were lower in patients with deactivation than in patients without deactivation, but there were no other differences between the two groups. During the 6 months before the last hospitalization, six (50%) of 12 patients with ICD deactivation and 23 (59%) of 39 without deactivation were hospitalized for worsening HF. During the last 3 months, five (42%) patients who deactivated ICD therapy and 16 (41%) who did not deactivate ICD therapy had received ICD shocks (*P* = .97) (Table 1).

**TABLE 1:** Characteristics of heart failure patients with and without deactivation of ICD

	Deactivation (+) (n = 12)	Deactivation (−) (n = 39)	*P*‐value
Age (y)	65 ± 4	66 ± 2	.93
Male	10 (83)	30 (77)	.63
NYHA functional class III/IV on last admission	6/6	15/24	.48
LVEF	33 ± 3	26 ± 2	.02
Underlying heart disease			.60
Coronary artery disease	1 (8)	7 (18)	
Nonischemic cardiomyopathies	10 (83)	27 (69)	
Valvular disease	1 (8)	2 (5)	
Others	0	3 (8)	
Heart transplant candidates	0	1 (3)	.58
SBP on last admission	89 ± 4	91 ± 3	.67
Plasma BNP (pg/mL)	936 ± 413	1502 ± 223	.23
eGFR (mL/min/1.73 m^2^)	33 ± 6	24 ± 3	.18
ICD indication			
Primary prevention	7 (58)	24 (62)	.84
Type of device			.38
Single‐chamber	4 (33)	6 (15)	
Dual‐chamber	2 (17)	10 (26)	
Biventricular	6 (50)	23 (59)	
Months from ICD implant to death	97 ± 62	72 ± 47	.15
ICD therapies for VT/VF within a year before death (ATP or shock)	7 (58)	17 (44)	.37
Electrical storm within a year of death	5 (42)	14 (36)	.71
Electrical storm within 30 d of death	4 (33)	8 (21)	.36
Any shock within a year of death (appropriate and inappropriate)	8 (67)	19 (49)	.47
Any shock within 90 d of death	5 (42)	16 (41)	.97
Any shock within 30 d of death	3 (25)	11 (28)	.83
Medications during the last hospitalization			
ACE inhibitors or ARBs	4 (33)	35 (90)	<.01
Beta blockers	7 (58)	35 (90)	.01
MRAs	7 (58)	29 (74)	.29
Diuretics	9 (75)	36 (92)	.10
Digoxin	7 (58)	15 (38)	.22
Amiodarone	7 (58)	28 (72)	.38
Intravenous inotropes	6 (50)	28 (72)	.16
Intravenous opioid	6 (50)	15 (38)	.48
Nonpharmacologic treatments during the last hospitalization			
NPPV	1 (8)	13 (33)	.09
Respirators	2 (17)	5 (13)	.73
IABP	3 (25)	13 (33)	.59
PCPS	1 (8)	5 (13)	.67

Note

Values are presented as numbers (%) or means ± SD.

Abbreviations: ACE, angiotensin‐converting enzyme; ARB, angiotensin II receptor blocker; ATP; antitachycardia pacing, BNP, brain natriuretic peptide; eGFR, estimated glomerular filtration rate; IABP, intra‐aortic balloon pumping; ICD, implantable cardioverter defibrillator; LVEF, left ventricular ejection fraction; MRA, mineralocorticoid receptor antagonist; NPPV, noninvasive positive pressure ventilation; NYHA, New York Heart Association; PCPS, percutaneous cardiopulmonary support; SBP, systolic blood pressure.

**FIGURE 4:**
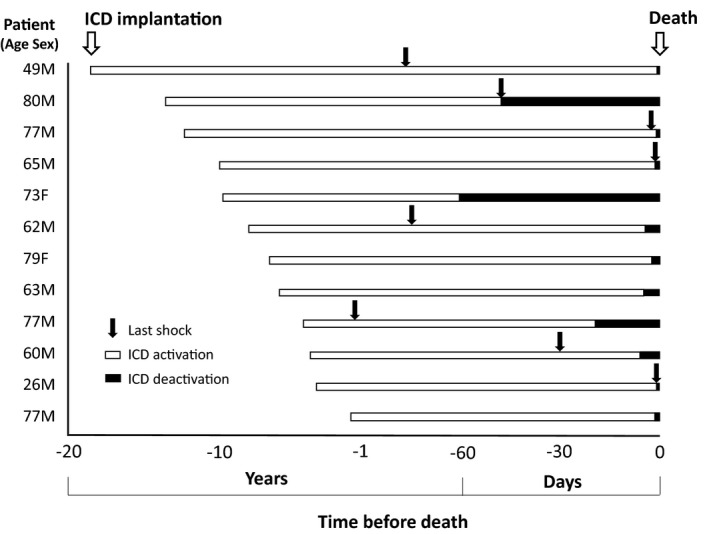
Implantable cardioverter defibrillator (ICD) implantation, last shock therapy, and deactivation before death for each of 12 patients who underwent ICD deactivation

The time course and events surrounding the end of life of each patient who underwent ICD deactivation are shown in Figure 4. All patients were NYHA class III/IV and had a history of several hospitalizations due to worsening HF. The median time from ICD implantation to death was 7.8 years, and the median time from deactivation to death was 3 days. ICD deactivation was performed within 1 week of death in eight (67%) patients. Although the time from the last shock to death ranged from 0 to 429 days, three patients experienced ICD shocks within a few days of death. Requests for ICD deactivation were mainly initiated by cardiologists (n = 9) followed by patients (n = 1) or family members (n = 2). The reasons for deactivation were as follows: avoidance of pain, unbearableness of frequent shocks, reduction of anxiety and a family member's being “unable to bear to watch the patient suffer”.

### 3.3 ICD therapy in patients with end‐stage HF

**FIGURE 5:**
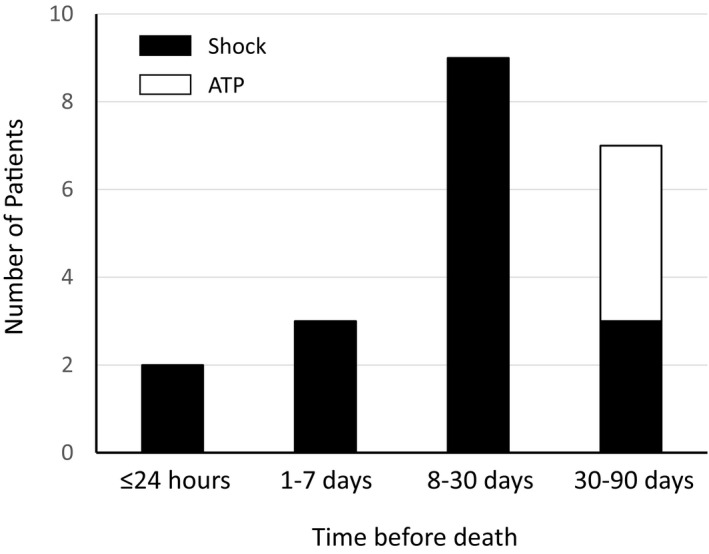
Frequency of patients experiencing implantable cardioverter defibrillator therapies 24 h, 1‐7 d, 8‐30 d, and 31‐90 d before death. ATP, antitachycardia pacing

The frequency of patients who received any ICD therapy (shock or ATP) is shown in Figure 5. The frequency of ICD therapy within 1 month of death was higher than that occurring earlier than 1 month of death in patients with end‐stage HF, and some patients received shocks within 24 hours of death. Among 39 patients without ICD deactivation, five patients received ICD shocks even after the DNAR decision. One patient who decided to deactivate ICD therapy received an ICD shock just before deactivation of the device and died 3 hours after deactivation.

## 4 DISCUSSION

Our study revealed the following findings: (a) 12 (24%) of 51 patients with end‐stage HF underwent ICD deactivation, and most of them received deactivation within 24 hours of the DNAR order; (b) 27 (69%) of 39 patients with DNAR orders did not undergo deactivation of ICD therapy; (c) 14 (27%) patients experienced ICD shocks within 1 month of death, and 12 (24%) experienced electrical storms within 1 month of death.

### 4.1 Deactivation of ICD therapy

Previous studies from the US and Canada showed that 15%‐33% of patients who were considered near the end of life deactivated ICD therapy.^5^, ^6^, ^8^, ^9^ These studies included patients with terminal conditions not only due to cardiac causes but also due to noncardiac causes. In our study, 24% of patients with end‐stage HF deactivated ICD therapy, which is comparable to these reports. However, only 12 (31%) of 39 patients with DNAR orders deactivated ICD therapy even after the DNAR order. Studies from Western countries reported that 49%‐100% of patients with DNAR orders deactivated ICD therapy.^5^, ^7^, ^9^ Different from the US, Canada and some European countries, there are currently no laws or guidelines regarding DNAR orders in Japan. Japanese physicians may fail to mention deactivation of ICD therapy in discussions of DNAR orders or palliative care for terminally ill patients. In our study, there was no documentation regarding ICD deactivation in discussions of DNAR for 23 of 27 patients who did not undergo deactivation of the ICD despite the DNAR order. The possible reasons for this were as follows: physicians and staff did not understand that ICD deactivation was included in the DNAR order; physicians believed that the patient would not experience ICD shock prior to death because ICD shock had not occurred in the past few months; and physicians might wish to allow the patient to die naturally of the underlying disease rather than terminate the patient's life due to the discontinuation of treatment. In the remaining four patients, one patient could not deactivate their ICD due to the rapid deterioration of their clinical condition, and the other patients decided to deactivate ICD if shock occurred after the DNAR order. The Heart Rhythm Society expert consensus statement states that discussion about device deactivation is essential during an ongoing process that starts when informed consent is obtained prior to ICD implantation and continues over time as the patient's condition and treatment goals change as the disease progresses.^13^

It may also be difficult to decide the timing of ICD deactivation in patients with end‐stage HF. In fact, one patient in our study experienced ICD shock in the period between the issue of a DNAR order and deactivation. Deactivation was performed within 24 hours after the DNAR decision in over half of the patients, but in other patients, the period until therapy deactivation was 2‐7 days or more. Physicians should recognize that the patients are at risk of shock therapy during this period. Of course, some patients may be unable to deactivate ICD treatment due to a rapid deterioration in their clinical condition. ^5^, ^6^ It may be preferable to deactivate ICD treatment promptly or at least within 24 hours after a DNAR order from the viewpoint of palliative medicine, which would promote the avoidance of painful shocks. Although permanent deactivation by reprogramming the device is desirable, temporally magnet application over the device generators is also acceptable in situations such as an urgent need for repetitive ICD shocks, in hospitals or other facilities lacking cardiologists or other professionals with electrophysiological expertise, or in home medical care, where individuals are not capable of reprogramming the ICD.^13^ In these cases, permanent deactivation should be scheduled to be performed by industry‐employed allied professionals within a few days, in collaboration with device manufacturers.

### 4.2 ICD therapy before death

In this study, 21 (41%) patients and 14 (27%) patients experienced ICD shock within 3 months and within 1 month of death, respectively; these represented higher frequencies than those patients who experienced ICD shock 3‐12 months before death. The frequency of electrical storms within 1 month of death was also high (24%). A subanalysis of MADIT‐II reported that eight (10%) of 83 patients without ICD deactivation experienced shock therapy within 1‐7 days of death, and 10 (12%) experienced ICD shock therapy within 24 hours of death.^6^ A Swedish observational study reported that the probability of shock treatment within 24 hours of death was 24% (95% CI, 11%‐37%).^7^ There was a high frequency of ICD therapy, including shock and ATP, within a month of death for patients with end‐stage HF. Worsening HF might contribute to the development of ventricular arrhythmias requiring ICD therapy. The problem of ICD deactivation in patients approaching death is also related to the high frequency of ICD shocks in the last weeks before death. Experience of ICD shock therapy when nearing death is also a factor in determining ICD deactivation. ^6^ In this study, even among patients with deactivation, three patients experienced ICD shock within a few days of death. From the viewpoint of palliative care, the avoidance of painful shocks is important when considering ICD deactivation and its timing.

### 4.3 Discussion of ICD deactivation

Requests for ICD deactivation were mainly initiated by cardiologists and, in some cases, patients or family members. Sherazi et al reported that a wide range of physicians, including cardiologists, intensive care specialists, and internal medicine residents as well as patients and families initiated requests for deactivation.^6^ The results of this study were related to focusing on patients with HF, but findings would be different in situations where other medical staff, such as nurses, also requested ICD deactivation. Palliative care of high‐risk patients with arrhythmias does not end with ICD deactivation, and beneficial pharmacologic therapy, including opioids, is recommended as appropriate symptomatic relief from dyspnea and pain for patients with end‐stage HF.^13^

Of course, advanced care planning for end of life in patients with ICDs does not mean the deactivation of ICD therapy. However, the condition of patients with cardiovascular disease may suddenly worsen even if the patient appears to be more stable. Decisions about ICD deactivation must be made considering the patient's condition, the risk of painful shock (risk of ICD) and the termination of life‐threatening arrhythmias (benefit of ICD).

In this study, patients who underwent deactivation of ICD therapy experienced frequent hospitalization due to worsening HF. For 15 years, our institution has held multidisciplinary conferences regarding patients with mental problems and patients with severe HF. Our multidisciplinary team included cardiologists, nurses/certified nurses in chronic HF nursing, psychiatrists, psychologists, pharmacists, social workers, and a rehabilitation team. In these meetings, we have discussed DNAR, including ICD deactivation. However, cases that were not raised for discussion in these conferences have usually been discussed between the attending cardiologist and the patient/family regarding DNAR; ICD deactivation has not always been included. Therefore, our results revealed that 27 patients did not undergo deactivation of the ICD despite the DNAR order, and the remaining 12 patients did not give a DNAR order. Advanced discussion regarding the deactivation of ICD therapy during the end of life might be necessary based on advanced care planning. To obtain the patient's stated or written advanced directive when the patient's condition is good would be helpful for discussion of ICD deactivation. Sherazi et al reported that most of the discussions regarding ICD deactivation were prompted by hospitalization for an acute deterioration of clinical status and by a change in the patients' advance directives and code status.^6^ When a patient with an ICD is approaching the end of life, discussion about ICD deactivation at the end of life is a core component of palliative care in HF and is also necessary in advanced care planning. This process will reduce the risk of receiving a painful shock and the experience of psychological distress, the latter applying not only to patients at the end of life but also to their family members.

Our study showed that only one‐fourth of patients with end‐stage HF underwent deactivation of ICD therapy. A relatively high frequency of shock therapy was observed within the last month of death. Discussions regarding ICD deactivation in palliative care are necessary to reduce the risk of painful shocks and distress during the end of life.

### 4.4 Study limitations

This study had several limitations. This study was a retrospective observational study performed at a single center. We did not evaluate the treatment and care status of ICD patients who died outside our hospital. The number of subjects was small. Therefore, the results of this study cannot be generalized to all patients with terminal illness. It should be noted that this study consisted of patients in the past decade. If advanced care planning for HF patients were to progress and guidelines for end‐of‐life care for noncancer patients were to become available in Japan in the future, the findings from this study would change. Because information regarding the discussion of and requests for ICD deactivation was obtained from medical records, the detailed context may be difficult to fully understand.

## CONFLICT OF INTEREST

The authors declare no conflicts of interest for this article.
